# Metabolic Basis and Clinical Evidence for Skin Lightening Effects of Thiol Compounds

**DOI:** 10.3390/antiox11030503

**Published:** 2022-03-04

**Authors:** Yong Chool Boo

**Affiliations:** 1 Department of Molecular Medicine, School of Medicine, Kyungpook National University, 680 Gukchaebosang-ro, Jung-gu, Daegu 41944, Korea; ycboo@knu.ac.kr; 2 BK21 Plus KNU Biomedical Convergence Program, Kyungpook National University, 680 Gukchaebosang-ro, Jung-gu, Daegu 41944, Korea; 3 Cell and Matrix Research Institute, Kyungpook National University, 680 Gukchaebosang-ro, Jung-gu, Daegu 41944, Korea

**Keywords:** melanin synthesis, eumelanin, pheomelanin, thiol compound, sulfhydryl compound, skin pigmentation, pigmentary disorder

## Abstract

Melanin pigment is a major factor in determining the color of the skin, and its abnormal increase or decrease can cause serious pigmentation disorders. The melanin pigment of the skin is divided into light pheomelanin and dark eumelanin, and a big difference between them is whether they contain sulfur. Melanin synthesis starts from a common reaction in which tyrosine or dihydroxyphenylalanine (DOPA) is oxidized by tyrosinase (TYR) to produce dopaquinone (DQ). DQ is spontaneously converted to leukodopachrome and then oxidized to dopachrome, which enters the eumelanin synthesis pathway. When DQ reacts with cysteine, cysteinyl dopa is generated, which is oxidized to cysteinyl DQ and enters the pheomelanin synthesis pathway. Therefore, thiol compounds can influence the relative synthesis of eumelanin and pheomelanin. In addition, thiol compounds can inhibit enzymatic activity by binding to copper ions at the active site of TYR, and act as an antioxidant scavenging reactive oxygen species and free radicals or as a modulator of redox balance, thereby inhibiting overall melanin synthesis. This review will cover the metabolic aspects of thiol compounds, the role of thiol compounds in melanin synthesis, comparison of the antimelanogenic effects of various thiol compounds, and clinical trials on the skin lightening efficacy of thiol compounds. We hope that this review will help identify the advantages and disadvantages of various thiol compounds as modulators of skin pigmentation and contribute to the development of safer and more effective strategies for the treatment of pigmentation disorders.

## 1. Introduction

Melanin is a colored biopolymer commonly found in most living organisms, and in humans, it is a major determinant of pigmentation of the skin, hair, mucous membranes, and retina [1,2,3,4]. Melanin synthesized in the melanosomes of epidermal melanocytes plays key roles in maintaining skin homeostasis [1,2], and photoprotection [5,6]. Skin pigmentary disease can be caused when melanin pigment is excessively high, low, or unevenly distributed [7,8,9]. The disease occupies an important part of skin problems, and medical demand is greatly increasing [10,11,12,13,14]. This disease is socially very important because it causes mental stress and reduces productivity and quality of life [15].

Prevention and treatment strategies for hyperpigmentation include photoprotection, topical therapy, surgical treatment (chemical peeling and laser treatment), and cosmetic camouflage [16,17]. Hydroquinone is used as primary therapy, usually in combination with retinoids or steroids, but it can cause side effects, such as skin irritation, allergies, mutations, and cancer [18,19]. Chemical peels and laser treatments are often performed, but there are side effects, such as dermatitis-induced pigmentation. In the cosmetic field, arbutin, nicotinamide (niacinamide), and ascorbic acid derivatives are mainly formulated for skin lightening effects [20,21,22].

Melanin is synthesized in melanosomes of melanocytes, and mature melanosomes filled with melanin are delivered to surrounding keratinocytes through the dendrites of melanocytes and spread to the skin [4,23]. Therefore, effectively controlling one of these multiple processes is a promising strategy to treat skin pigmentation. The most well studied molecular targets for artificially regulating skin pigmentation are (1) the receptors on the surface of melanocytes that transmit intracellular signals, (2) the enzymes and proteins involved in melanin synthesis in melanosomes, and (3) the biogenesis, maturation, and intercellular transfer of melanosomes [24]. This research team discovered and reported glycinamide and low molecular weight peptides that act on the first target and exhibit skin depigmenting effects in previous studies [25,26]. In addition, the skin depigmenting effects of resveratrol derivatives and p-coumaric acid acting on the second target have also been reported [27,28].

Melanin synthesis starts from a common reaction in which tyrosine or dihydroxyphenylalanine (DOPA) is oxidized by tyrosinase (TYR) to produce dopaquinone (DQ) [29], which enters either the eumelanin (brownish-black color) or pheomelanin (reddish-yellow color) synthesis pathway depending on the availability of thiol compounds, such as cysteine and glutathione [4,30,31]. The relative content of eumelanin and pheomelanin has been associated with skin color [32]. Human skin color and glutathione content are correlated with each other [33]. In Tortoiseshell guinea pigs, the content of reduced glutathione was lower in the black skin area than in the red or yellow skin area [34]. Thus, the use of thiol compounds that can affect the relative synthesis of eumelanin versus pheomelanin is a promising strategy to control skin pigmentation. Furthermore, several thiol compounds can affect cellular redox homeostasis, leading to changes in cellular melanogenesis and skin pigmentation.

In this review, we will first summarize metabolic processes involving thiol compounds, such as cysteine, glutathione, and coenzyme A. We will then discuss the effects of different thiol compounds on in vitro tyrosinase enzyme activity and melanogenesis at the cellular level. Additionally, we will discuss clinical trials on the skin lightening efficacy of thiol compounds. It is hoped that this review will help understand the advantages and disadvantages of thiol compounds as modulators of skin pigmentation and contribute to the development of safer and more effective treatment strategies for pigmentation disorders.

## 2. The Metabolism of Cysteine and Related Thiol Compounds

**Figure 1:**
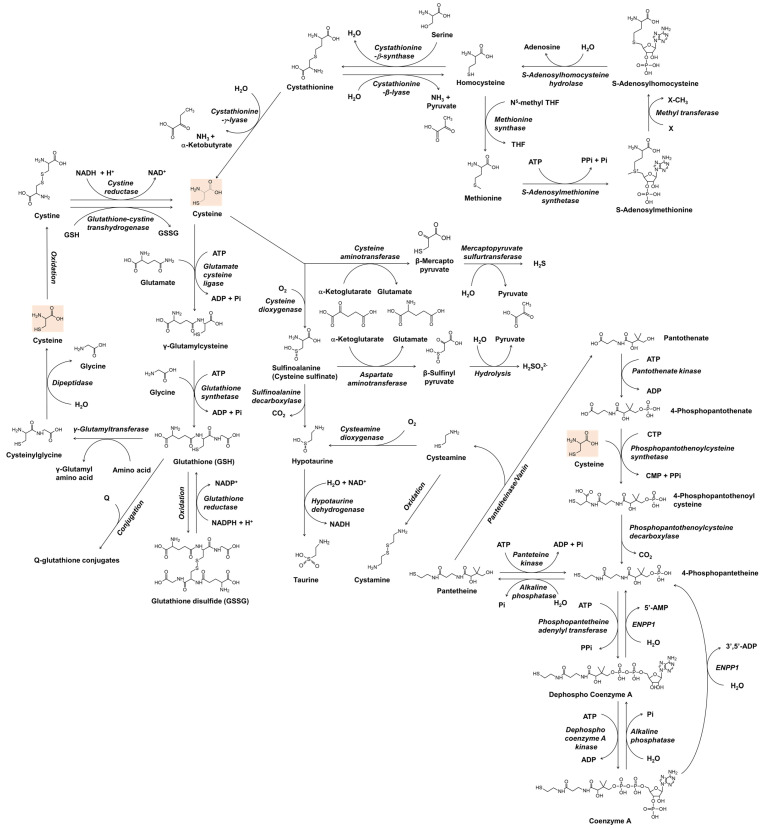
The metabolism of cysteine and related thiol compounds in mammals. ADP, adenosine diphosphate; AMP, adenosine monophosphate; ATP, adenosine triphosphate; CDP, cytidine diphosphate; CTP, cytidine triphosphate; ENPP1, ectonucleotide pyrophosphatase/phosphodiesterase 1; GSH, glutathione; GSSG, glutathione disulfide; NAD^+^, nicotinamide adenine dinucleotide; NADH, nicotinamide adenine dinucleotide hydrogen; NADP^+^, nicotinamide adenine dinucleotide phosphate; NADPH, nicotinamide adenine dinucleotide phosphate hydrogen; Pi, inorganic phosphate; PPi, inorganic pyrophosphate; Q, substrates or reactants for glutathionylation; THF, tetrahydrofolate; X, substrates for methylation.

Before understanding the role of thiol compounds in melanogenesis, basic knowledge of the metabolism of these compounds is required. Figure 1 shows the anabolic and catabolic pathways of cysteine and glutathione that have been studied as skin lightening cosmeceuticals. The metabolic pathway of coenzyme A is also included because cysteamine, which is produced during its catabolism, has been developed as a skin lightener. Understanding the interconnected metabolism will help to predict the direct and indirect biological effects of a certain thiol compound and its potential side effects. In Figure 1, only the reactions of enzymes whose existence has been known in mammals are shown. The general information for each enzyme is available from BRENDA enzyme database at https://www.brenda-enzymes.org (accessed on 3 March 2022).

In bacteria and plants, *O*-acetyl serine, an activated form of serine, reacts with sulfide sources under cysteine synthase catalyst to produce cysteine and acetate [35,36]. In mammals, cysteine is made from serine, and the source of sulfur is methionine [37]. Methionine is converted to homocysteine via *S*-adenosyl methionine and *S*-adenosyl homocysteine [38]. Cystathionine β-synthase combines homocysteine with serine to form asymmetrical thioether cystathionine, and this compound is cleaved by cystathionine γ-lyase into α-ketobutyrate, ammonia, and cysteine [39]. If cystathionine is cleaved by cystathionine β-lyase, it produces pyruvate, ammonia, and homocysteine [40]. Homocysteine can be converted to methionine by methionine synthase, which uses N^5^-methyl tetrahydrofolate as a methyl group donor [41].

In addition to its biosynthesis in situ in the cells, cysteine and its oxidized form cystine are imported from the outside of the cells to meet the cells’ high metabolic needs. Extracellular cysteine is readily oxidized and exists mainly in the form of cystine [42]. Cystine transport is mainly performed by the cystine/glutamate transporter, known as system Xc- or xCT, which is encoded by the solute carrier family 7 member 11 (SLC7A11) gene in humans [43]. It is an antiporter that imports cystine and exports glutamate [44]. Once cystine is transported across the membrane into cells, it is reduced back to cysteine by cystine reductase or glutathione-cystine transhydrogenase [45,46]. Cysteine itself can also be transported into the cell by other transporters, such as excitatory amino acid transporter 3 (EAAT3) [47]. Recently, major facilitator superfamily domain containing 12 (MFSD12) has been reported to transport cysteine to melanosomes or lysosomes [48].

Cysteine is used not only as a building block of proteins but also in the synthesis of various thiols that play crucial roles in maintaining redox homeostasis and cellular metabolism [49]. Glutathione is a tripeptide with multiple functions associated with antioxidant defense and other related cell physiology [50,51]. For the synthesis of glutathione, cysteine is first combined with glutamate to form γ-glutamyl cysteine, and then glycine is added to synthesize glutathione [52]. Glutathione can be oxidized to glutathione disulfide by several oxidoreductases, such as glutathione peroxidase, or non-enzymatically by various reactive oxygen species (ROS) or oxidizing agents [53]. Glutathione disulfide is reduced in a glutathione reductase-catalyzed reaction, in which nicotinamide adenine dinucleotide phosphate hydrogen (NADPH) is consumed [54]. Glutathione also binds to substrates in the metabolic process of xenobiotics to form conjugates [55]. Glutathione, its oxidized form, and its conjugates can be exported out of the cell by specific transporters [56]. At the cell surface, the glutamyl moiety of glutathione is transferred to other amino acids by γ-glutamyl transpeptidase, and cysteinyl glycine is produced [57]. Cysteinyl glycine is further cleaved into cysteine and glycine by dipeptidase, and these free amino acids can enter the cell by specific transporters [58].

Coenzyme A is an essential cofactor for many enzymes that mediate more than 100 different catabolic and anabolic reactions of lipids, carbohydrates, proteins, ethanol, bile acids, and xenobiotics [59]. Cysteine enters the synthesis pathway of coenzyme A by reacting with 4-phosphopantothenate to produce 4-phosphopantothenoyl cysteine [60]. 4-Phosphopantetheine, which is produced by decarboxylation of 4-phosphopantothenoyl cysteine, or by phosphorylation of pantetheine, is converted to coenzyme A through two steps of ATP-dependent phosphorylation [61]. In the catabolic pathway of coenzyme A, dephosphorylation by ectonucleotide pyrophosphatase/phosphodiesterase 1 (ENPP1) produces 4-phosphopantetheine, and further dephosphorylation produces pantetheine [62]. Pantetheine is cleaved by pantetheinase/vanin to form cysteamine and pantethenate [63]. Cysteamine is not only a metabolite biosynthesized in cells but also a drug candidate with various therapeutic potentials [64,65]. Cysteamine is oxidized to cystamine dimer or reacted with oxygen in a cysteamine dioxygenase catalyzed reaction to form hypotaurine [65]. Taurine, which is produced in the oxidation reaction of hypotaurine, is a non-protein amino acid that regulates various cellular functions, including osmoregulation, ion movement modulation, bile acid conjugation, antioxidation, anti-inflammation, and energy metabolism regulation [66,67].

In its catabolic pathways, cysteine undergoes transamination to form β-mercaptopyruvate, which is enzymatically decomposed into pyruvate and hydrogen sulfide [68]. Hydrogen sulfide produced in many enzymatic reactions affects various cell functions, and various therapies using it are also being studied [69]. In another catabolic pathway, cysteine is oxidized to sulfinoalanine (cysteine sulfinate) by the action of cysteine dioxygenase [70]. Sulfinoalanine is decarboxylated and leads to the formation of hypotaurine and taurine in a sequence, or is transaminated and produces β-sulfinyl pyruvate, which is enzymatically or spontaneously hydrolyzed to pyruvate and sulfite [71].

## 3. The Role of Thiol Compounds in Melanin Synthesis

Microphthalmia-associated transcription factor (MITF) regulates gene expression of melanogenic enzymes such as TYR, tyrosinase-related protein 1 (TYRP1), and dopachrome tautomerase (DCT) in response to a variety of internal and external stimuli [72,73]. α-Melanocyte stimulating hormone/melanocortin 1 receptor/adenyl cyclase/cyclic adenosine monophosphate (cAMP)/protein kinase A/cAMP-reactive element-binding protein pathway activates MITF [72,73]. In addition, the stem cell factor/receptor tyrosine kinase c-Kit/mitogen-activated protein kinase pathway and the Wingless-related integration site (WNT)/frizzled/glycogen synthase kinase 3β/β-catenin pathway activate MITF [74,75]. Other signaling pathways, such as the phospholipase C/diacylglycerol/protein kinase Cβ cascade and the nitric oxide/cyclic guanosine monophosphate (cGMP)/protein kinase G cascade also activate MITF [76,77]. Please refer to other comprehensive reviews on autocrine and paracrine regulation of melanogenesis [77,78].

**Figure 2:**
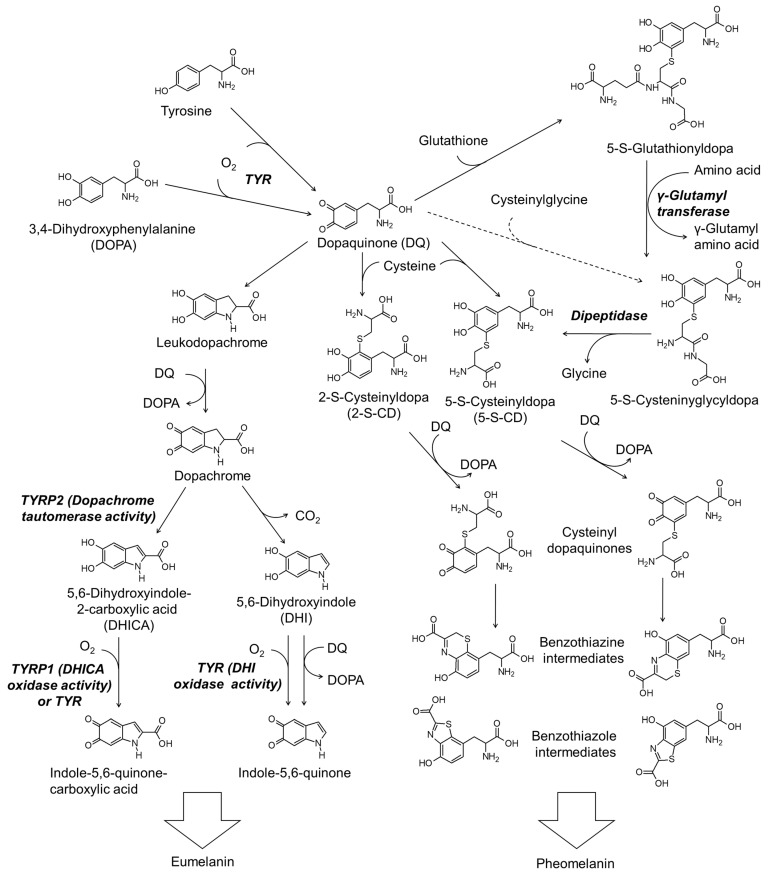
The biosynthetic pathways for eumelanin and pheomelanin. TYR, tyrosinase; TYRP1, tyrosinase-related protein 1; TYRP2, tyrosinase-related protein 2.

Figure 2 shows the simplified scheme of the biosynthesis pathway for eumelanin and pheomelanin. TYR catalyzes the initial step of melanin synthesis, which is the oxidation of tyrosine or DOPA to DQ [29]. The subsequent reactions differ depending on the availability of thiol compounds [31].

When the thiol compound is deficient, DQ is spontaneously cyclized to leukodopa, which is further oxidized to dopachrome in concert with the reduction of DQ to DOPA [79]. Dopachrome is then tautomerized to form 5,6-dihydroxyindole-2-carboxylic acid (DHICA) or decarboxylate to form 5,6-dihydroxyindole (DHI) [80]. DHICA and DHI undergo oxidation to form their quinones enzymatically or non-enzymatically [81]. Polymerization of DHI and DHICA and their quinones leads to the formation of eumelanin [76].

Thiol compounds such as cysteine and glutathione rapidly react with DQ produced in the TYR-catalyzed reaction [82]. If cysteine is the reactant, 5-*S*-cysteinyl dopa (5-*S*-CD) or 2-*S*-cysteinyl dopa (2-*S*-CD) are produced [83]. 5-*S*-CD and 2-*S*-CD are then oxidized to their quinones in concert with the reduction of DQ to DOPA or in a TYR-catalyzed reaction [84] and then cyclized to benzothiazine or benzothiazole intermediates [30,85]. The polymerization reaction between these intermediates leads to the production of the final product, pheomelanin [4,30,31].

Glutathione can react with DQ to produce 5-*S*-glutathionyl dopa and 2-*S*-glutathionyl dopa in preference of the former [86]. 6-*S*-Glutathionyl dopa is also formed at a lower yield (5%) compared to 5-*S*-glutathionyl dopa (76%) and 2-*S*-glutathionyl dopa (12%) [86]. Carstam et al. showed that 5-*S*-glutathionyl dopa is present in the homogenates of cultured human melanoma cells at a hundred times lower molar concentration than that of 5-*S*-cysteinyl dopa [87]. They also detected 5-*S*-cysteinyl glycyl dopa at a ten times lower level, but γ-glutamyl-5-*S*-cysteinyl dopa was not detected. Thus, 5-*S*-glutathionyl dopa is thought to be converted to 5-*S*-cysteinyl glycyl dopa by γ-glutamyl transpeptidase, and then to 5-*S*-CD by dipeptidase. 2-*S*-Glutathionydopa may also be converted to the respective 2-*S*-CD through a similar process. Thus formed 5-*S*-CD and 2-*S*-CD undergo oxidation to their quinones and subsequent cyclization to the benzothiazine or benzothiazole intermediates, as described above.

The possibility that cysteinyl glycine produced during the decomposition of glutathione directly reacts with DQ to produce 5-*S*-cysteinyl glycyl dopa cannot be excluded. However, the possibility that γ-glutamyl cysteine produced during the synthesis of glutathione reacts with DQ to produce γ-glutamyl-5-*S*-cysteinyl dopa seems to be insignificant. Under the circumstance when the syntheses of glutathione and cysteinyl dopa competitively consume cysteine, an increase in the synthesis of glutathione can cause a decrease in the production of cysteinyl dopa, and vice versa [88].

The melanogenesis occurring in melanosomes can be affected by various factors including the concentration of thiol compounds and other metabolites, oxygen partial pressure, and pH in this organelle. In particular, the metabolism of glutathione is predicted to maintain a dynamic parallel relationship with the synthesis of pheomelanin, sometimes in a cooperative relationship and sometimes in competition.

## 4. TYR-Inhibitory and Antimelanogenic Effects of Various Thiol Compounds

### 4.1. TYR-Inhibitory Effects In Vitro

TYR oxidizes pyrocatechol to ortho-quinone, and when thiol compounds are present, a pyrocatechol-cysteine conjugate is generated [89]. Many studies have used colorimetric methods monitoring the production of dopachrome from tyrosine or DOPA to measure the activity of TYR. Therefore, it is difficult to distinguish whether the test substance directly inhibits the catalytic activity of the enzyme to prevent the production of DQ, or traps the reaction product, DQ, to prevent its oxidation to dopachrome. For most thiol compounds, it is considered that the latter action takes precedence over the former action. With this point in mind, we here discuss the studies reporting the inhibitory effects of various thiol compounds on the TYR-catalyzed reaction.

Kahn et al. compared the effects of various amino acids on the dihydroxyphenolase activity of mushroom TYR using DOPA as a substrate [90]. Cysteine exhibited the most potent TYR-inhibitory effect, extending an initial delay (lag period) in dopachrome formation and suppressing it completely at 0.3 mM. In Jara et al.’s study, cysteine inhibited mouse melanoma TYR hydroxylase activity measured by radioactive water released from L-[3,5-^3^H]-tyrosine substrate (50% inhibitory concentration (IC_50_), 0.66 mM), as well as the DOPA oxidase activity of TYR measured by dopachrome formation [82]. In addition, cysteine at 0.15 or 0.30 mM inhibited mushroom TYR activity measured by a spectrophotometric method (IC_50_, 0.15 mM) and a polarographic method (IC_50_, 1.44 mM) [91]. Therefore, cysteine is one of the amino acids whose reactivity is unique, and it can directly inhibit the activity of TYR in addition to reducing the production of dopachrome.

The two Cu^2+^ ions are present at the active site of the TYR enzyme, each being coordinated by three histidine residues [92]. In the study of Jergil et al., cysteine at high concentrations (10 mM) inactivated TYR, whereas the addition of tyrosine and DOPA competitively restored the enzyme activity [93]. It is presumed that cysteine can bind to copper at the active site of the TYR, inactivating the enzyme, and the substrates block cysteine’s access to the active site of the enzyme.

Tseng et al. compared the inhibitory effects of 20 × 20 dipeptides against mushroom TYR and found that cysteine-containing dipeptides are potent inhibitors and that *N*-terminal cysteine-containing dipeptides are more potent inhibitors than *C-*terminal cysteine-containing dipeptides [94]. In a study by Hsiao et al., cysteine-containing tripeptides, such as arginine-cysteine-tyrosine and cysteine-arginine-tyrosine, exhibited potent inhibitory effects against mushroom TYR activity [95]. Cysteine-arginine-tyrosine tripeptide containing a cysteine residue at its *N*-terminus was estimated to be a more potent TYR inhibitor (IC_50_, 6.16 μM) compared with kojic acid (IC_50_, 84.4 μM) and arbutin (IC_50_, 1008.7 μM). These studies suggest that the *N*-terminal cysteine residue of certain peptides reacts faster with DQ than others. It also suggests a possibility that cysteinyl glycine, a product of the degradation process of glutathione, may react more rapidly with DQ than does γ-glutamyl cysteine, a substrate of the synthesis process of glutathione.

TYR inhibitory effects of a series of sulfurated amino acids and tripeptides were compared by Luisi et al. [96]. The results showed that cysteine, cystine, γ-oxa-glutamyl analog of glutathione, and ergothioneine exhibited more potent inhibition compared with glutathione, whereas taurine exhibited a weaker inhibition.

In a recent study, we compared the effects of twenty different amidated amino acids on human TYR-mediated dopachrome formation in vitro [97]. The results showed that only cysteinamide among the tested amidated amino acids inhibited the DOPA formation effectively. Its inhibitory effect against TYR-mediated dopachrome formation was superior to those of cysteine, *N*-acetyl cysteine, glutathione, kojic acid, and arbutin. We have proposed that cysteinamide may attenuate eumelanin synthesis through a dual mechanism by diverting DQ to the formation of DOPA-cysteinamide conjugates (at 200 μM) and directly inactivating the enzyme through chelation of copper at the active site of the TYR at higher concentrations (500 μM) [97].

These studies show that thiol compounds may vary in their inhibitory effects on the activity of TYR or their reactivity with DQ depending on their chemical structure. In addition, although many studies have used mushroom TYR as an alternative for the human enzyme, these enzymes are quite different in amino acid sequence and substrate specificity [98,99]. Human and mushroom TYRs are inhibited differently by an identical compound [100,101]. Therefore, the inhibitory effect of a certain compound on mushroom TYR cannot be applied directly to human enzymes, and there is a limit to predicting its effect on cellular melanin synthesis based on in vitro enzyme assay results.

### 4.2. Anti-Melanogenic Effects in Cells

Deprivation of cysteine induced an increase in eumelanin synthesis as seen in human melanoma cells [102]. Conversely, supplementation of cysteine increases the pheomelanin/total melanin ratio in melanocytes, especially in a high tyrosine medium [103]. Therefore, the concentration and the relative ratio of tyrosine and cysteine will affect the concentration of total melanin and the ratio of eumelanin and pheomelanin synthesized.

Qiu et al. compared the effects of various thiol compounds on cell melanin content at their maximally tolerated concentrations in human melanoma cells (MM418c5) [104]. As a result, different thiol compounds showed varied melanin-reducing effects: dithiothreitol, 98% reduction at 650 μM; phenyl thiourea, 97% reduction at 200 μM; cystamine, 95% reduction at 50 μM; cysteamine, 79% reduction at 100 μM; cysteine, 7% reduction at 1 mM; glutathione, 5% reduction at 1 mM. Chung et al. examined the effects of glutathione and its derivatives, such as glutathione ethyl ester, glutathione diethyl ester, and glutathione isopropyl ester, on the melanin production in Melan-A cells [105]. The results showed that glutathione itself had no inhibitory effect on melanin production, but glutathione ethyl ester increased pheomelanin content and the pheomelanin/eumelanin ratio without significant effects on the expression of MITF, TYR, TYRP1, and DCT.

Choi et al. discovered the antimelanogenic effects of a homodimer of dipeptides containing cysteine and methionine residues connected by an intramolecular disulfide bond [106]. The peptide did not affect TYR catalytic activity in vitro but reduced melanin production in normal human melanocytes by suppression of MITF and downregulation of TYR protein.

In our recent study [97], cysteinamide at 1 mM exhibited a more potent antimelanogenic effect compared to other thiol compounds, such as cysteine, *N*-acetyl cysteine, glutathione, cysteine ethyl ester, *N*-acetyl cysteinamide, and cysteamine, as well as other TYR inhibitors, such as kojic acid, and β-arbutin at the same concentration in MNT-1 cells. Cysteine ethyl ester, arbutin, and cysteamine exhibited cytotoxicity in order. Cysteinamide exhibited comparable inhibitory effects against melanin synthesis in normal human epidermal melanocytes without altering the mRNA levels of TYR, TYRP1, and DCT.

Therefore, the potency of biological activity and mechanism of action of thiol compounds in inhibiting cell melanin synthesis will vary depending on the chemical structure of individual compounds. It should be noted that the response of malignant melanocytes used in several studies may not necessarily be the same as that of normal melanocytes. In addition, although the results of experiments using normal human melanocytes are relatively more reliable, verification of the depigmenting efficacy in clinical studies is ultimately required.

## 5. Clinical Trials on the Skin Lightening Efficacy of Thiol Compounds

### 5.1. Glutathione and Glutathione Disulfide

Glutathione is an important component to maintain intracellular redox balance and regulates cell melanin production through various mechanisms, so its skin lightening effect was expected [107]. The results of clinical trials on the skin lightening effect and safety of glutathione reported so far are not fully consistent or conclusive [108,109].

A clinical study on the skin lightening action of reduced glutathione was reported early in the 1960s in Japan [110]. After oral administration of glutathione, facial pigmentation disorders in women were significantly improved. According to a clinical study reported in Korea in 1977, out of 150 patients with melasma who took 50–100 mg of glutathione orally three times a day for 6 weeks on average, 74.1%, 56.7%, 7.3%, and 18.6% showed excellent, good, fair, and no improvement, respectively [111].

Table 1 shows selected clinical studies that were conducted to evaluate the skin lightening efficacy of glutathione and glutathione disulfide. Arjinpathana et al. [112] reported that when glutathione was taken orally, the symptoms of melasma and the appearance of spots caused by UV exposure were reduced [112]. Because glutathione is easily oxidized, it is difficult to apply in cosmetic formulations. On the other hand, glutathione disulfide, an oxidized form of glutathione, is relatively stable and can regenerate reduced glutathione in cells. Based on these ideas, Watanabe et al. evaluated the skin lightening efficacy of topical application of glutathione disulfide [113]. As a result, the skin melanin index was significantly reduced compared to the placebo. Glutathione disulfide also showed the effect of increasing skin moisture and reducing wrinkles [113]. In a study by Weschawalit et al., both oral administrations of glutathione and glutathione disulfide reduced the melanin index and UV spots compared to the placebo group [114]. These studies suggest that both glutathione and its oxidized form can give beneficial effects in alleviating hyperpigmentation.

Oral administration of glutathione has a problem in that it disappears through the gastrointestinal tract, and topical administration has a disadvantage in that it is difficult to absorb into the skin. To avoid these problems, direct intravenous injection of glutathione and absorption through the oral mucosa have been attempted. Intravenous injection of glutathione showed a remarkable skin lightening effect, various side effects appeared in almost all patients, and there was a problem that the therapeutic effect disappeared within a few months after the end of treatment [115]. In a clinical trial in which glutathione contained in a lozenge was absorbed through the mucous membrane in the mouth, a skin lightening effect was observed without major side effects [116]. However, the superiority of this method was not demonstrated because it was not directly compared with oral administration.

In a recent clinical study, Duperray et al. evaluated the skin lightening effect in patients who were orally administered glutathione 250 mg, cystine 500 mg, or glutathione 250 mg plus cystine 500 mg [117]. Overall, the skin lightening effect in all groups was not large, but in the group treated with glutathione or cysteine alone, the lightness and the individual typology angles (ITA°) of certain skin sites were significantly higher than those of the placebo group. ITA° values are calculated using the equation: ITA° = (arc tangent [(L* − 50)/b*]) 180/3.14159 [118], and higher ITA° value represents lighter skin color. Notably, the group treated with glutathione and cystine together exhibited the most excellent skin lightening effect, and there were significant differences in skin lightness and ITA^o^ values compared to the group treated with glutathione or cystine alone as well as the placebo group.

### 5.2. Cysteine, N-Acetyl Cysteine and Cystine

*N*-acetyl cysteine is a donor of cysteine and is being extensively studied for dermatological applications [119]. Its effect against human melasma has been examined in a double-blind, placebo-controlled, split-face study [120]. Melasma patients applied a cream containing 4.7% *N*-acetyl cysteine and 2% hydroquinone on one side of the face and the cream base on the other side for 4 months. Mild-to-strong bleaching of the skin was observed where an active cream was applied in 9 out of 10 female patients. The other side where the cream base was applied showed no significant changes in these 9 patients. The effect of cysteine alone was not evaluated in this trial.

Changes in skin luminosity score due to UV-B exposure (0.384 J cm^−1^, on day 8, 10, 12, 15, 17, and 19) were monitored in brown guinea pigs orally administered either ascorbic acid (600 mg kg^−1^) alone or in combination with cysteine (160 mg kg^−1^) and α-tocopherol (50 mg kg^−1^) daily for approximately 5 weeks [121]. The skin luminosity score of the triple combination group was higher than that of the control group, ascorbic acid alone group, and ascorbic acid plus cysteine group (n = 12, each group). There was no evidence of the skin lightening effect of cysteine itself in this animal study.

As mentioned in the above section, in a clinical trial, a skin lightening effect was observed after a combination of glutathione 250 mg plus cystine 500 mg was orally administered, and a weaker but significant skin lightening effect was observed when only 500 mg of cystine was administered [117]. Overall, clinical validation of the skin lightening effects of cysteine, *N*-acetyl cysteine, and cystine is still insufficient to draw any conclusion, and additional research is needed.

### 5.3. Cysteamine

Numerous clinical trial results have been reported on the skin lightening efficacy of cysteamine [122], and selected studies are shown in Table 2.

Cysteamine 5% cream significantly improved melasma area severity index (MASI) and the investigator’s global assessment (IGA) and reduced the degree of pigmentation measured by Mexameter and Dermacatch compared to placebo [123,124]. The melasma improvement efficacy of cysteamine 5% cream was comparable to that of the modified Kligman’s formula (MKF) comprising 4% hydroquinone, 0.05% retinoic acid, and 0.1% betamethasone [125]. Cysteamine cream showed relatively better skin tolerability than MKF. Cysteamine 5% cream showed efficacy similar to that of hydroquinone 4% cream in the treatment of melasma, and the latter showed relatively good skin tolerability [126]. In a clinical trial comparing the efficacy of the application of cysteamine cream and the mesotherapy of tranexamic acid, the modified MASI and the IGA were decreased to a similar extent in both groups [127]. Therefore, although there is a debate about the skin tolerability of cysteamine, its skin lightening effect is evaluated to be significant.

## 6. Discussion

The metabolism of thiol compounds such as cysteine and glutathione is closely related to the synthesis of melanin, especially the relative synthesis of eumelanin and pheomelanin. When cysteine is sufficient, glutathione synthesis occurs well, and cysteine and glutathione rapidly quench DQ to generate dopa-thiol conjugates. As a result, it will increase the production of pheomelanin rather than eumelanin. However, when cysteine is insufficient, the syntheses of glutathione and cysteinyl dopa compete for the limited cysteine, and the possibility of eumelanin synthesis is higher than that of pheomelanin. This scenario has been the metabolic basis for a strategy to pursue skin lightening using thiol compounds.

What happens if the concentration of cysteine is too high? A very high concentration of cysteine chelates copper at the active site of TYR, inactivates the enzyme, and inhibits the synthesis of both eumelanin and pheomelanin, leading to extreme hypopigmentation. At this time, the activity of other metal-containing enzymes in the cell also can be inactivated, which will disturb overall cell physiology. Interestingly, it was reported that increased homocysteine levels were associated with hypopigmentation disorders [128,129]. Serum homocysteine levels are highly elevated in vitiligo patients [130]. The ROS produced by homocysteine oxidation might induce the apoptosis of melanocytes [131]. It was also reported that homocysteine inactivated the TYR enzyme by binding copper at the active site of the enzyme [132]. Thus, an abnormal increase in local homocysteine concentration can inhibit normal melanogenesis and survival of melanocytes, causing the pathogenesis of vitiligo. In support of this notion, the expression level of *S*-adenosylhomocysteine hydrolase producing homocysteine was upregulated in the vitiliginous skin [133], and its specific inhibitor 3-deazaneplanocin A increased melanin synthesis in B16/F10 murine melanoma cells [134].

To reduce the synthesis of eumelanin and to increase the synthesis of pheomelanin, a safe and effective nutrient source capable of increasing the pool of thiol compounds in cells is required. According to the results of our study [97], 1 mM of cysteinamide effectively inhibited cell melanogenesis compared to the same concentration of cysteine and other thiol compounds, such as *N*-acetyl cysteine, glutathione, cysteine ethyl ester, and *N*-acetyl cysteinamide. Cysteinamide increased pheomelanin content while decreasing total melanin and eumelanin content in melanocytes. This suggests that cysteinamide has advantageous properties that can safely and efficiently increase the thiol pool of cells and enhance pheomelanin synthesis.

Cysteine supplied from the outside enters the cell through the cystine/glutamate antiporter mainly in the form of oxidized cysteine [43,44]. It can also enter the cells in the form of cysteine through other transporters [47]. Cysteine can be transported to melanosomes or lysosomes via recently identified MFSD12 [48]. On the other hand, it is not known how cysteinamide gets into cells and melanosomes. Further studies are needed to determine which systems are involved in the transport of cysteinamide and whether cysteinamide is oxidized or converted to other metabolites during its transport process.

Cellular melanin synthesis can be regulated not only by inhibiting the enzymatic reaction but also by suppressing the mRNA and protein expression of melanogenic enzymes. Cysteinamide decreased melanin synthesis without affecting the mRNA and protein expression levels of TYR, TYRP1, and DCT, so it appears to act through the former mechanism [97]. On the other hand, a homodimer of dipeptides tested in the study of Choi et al. appears to reduce melanin through the latter mechanism of suppressing MITF and TYR protein expression without affecting TYR catalytic activity in vitro [106].

Changes in cellular redox balance also affect melanin synthesis [20]. External factors, such as ultraviolet (UV) rays, can stimulate melanin synthesis by increasing the production of reactive oxygen species (ROS) in cells [135]. Melanin acts to block UV rays in the skin [136], but its synthetic process generates ROS and free radicals [137]. ROS from various sources can affect the melanogenic activity of melanocytes, causing hyperpigmentation or hypopigmentation [135]. Thiol compounds can reduce melanogenesis by acting as an antioxidant scavenging ROS and free radicals or as a modulator of redox balance [138].

In clinical trials, the effects of glutathione and its oxidized form, cysteamine, cysteine, and its oxidized form, alone or in combination, administered by various methods, such as transdermal, oral, and intravenous administration, on skin color, number of UV spots, and severity of melasma were investigated. The oral or topical application of thiol compounds did not have any notable side effects at the concentrations used in most cases. On the other hand, when glutathione was administered via intravenous injection, adverse reactions of varying severity were observed in all patients, requiring special attention. The tested thiol compound in general showed significant improvement in skin color, UV spots, and melasma compared to pre-use and placebo. Interestingly, the reduced and oxidized forms of thiol compounds show similar efficacy, which seems to be reasonable because they are readily interconvertible inside and outside the cell.

Challenges to overcome for topical application of thiol compounds to the skin include enhancing stability and promoting transdermal absorption [139,140]. Since thiol compounds have a characteristic odor and their degradation products give off an irritating odor, the development of a special formulation is highly required. Nevertheless, the value of thiol compounds as a skin lightener that can replace hydroquinone, which is of concern regarding toxicity in cosmetics where safety is important, needs to be reevaluated. Many plants, such as asparagus, contain cysteine, *N*-acetyl cysteine, homocysteine, γ-glutamyl cysteine, and glutathione in substantial concentrations, so the extracts of these plants can be used as a resource for thiols [141]. In addition, numerous thiol compounds of microbial and marine origin, such as coenzyme B, coenzyme M, and mycothiol, have been reported [142]. It is necessary to study whether these uncommon thiol compounds can be used in treating hyperpigmentation or hypopigmentation disorders.

## 7. Conclusions

In this review, the metabolism of thiol compounds was examined with a focus on the synthesis and degradation of cysteine, glutathione, and coenzyme A. In addition, it was discussed that the synthesis of pheomelanin can compete or cooperate with the synthesis of glutathione depending on the availability of cysteine. Thiol compounds have common properties useful as modulators of melanin synthesis and skin pigmentation. However, the actual mechanism of action and clinical efficacy depend on the individual chemical structures of various thiol compounds.

Several clinical trials have been conducted on the skin lightening effects of glutathione, its oxidized form, and cysteamine, and the evidence for their efficacy has been partially established. Clinical trials on cysteine, *N*-acetyl cysteine, and cystine are still insufficient. Cysteinamide, which has been found to more safely and effectively inhibit the production of eumelanin in cells compared to other thiol compounds, is expected to open up new possibilities for skin lightening. Further research and clinical trials are needed to verify the merits of cysteinamide as a depigmenting cosmeceutical compared to other thiol compounds, such as cysteine, glutathione, and cysteamine.

## Figures and Tables

**Table 1:** Clinical trials on the skin lightening efficacy of glutathione and glutathione disulfide.

Literature	Study Format	No. of Subjects	Tested Materials	Treatment	Key Findings
[112]	A double-blind, randomized, placebo-controlled study	30	Placebo capsules	The capsules were orally taken twice daily for 4 weeks.	Compared to the baseline values, the melanin indices and the number of ultraviolet (UV) spots at all six skin sites decreased consistently in subjects who received glutathione for 4 weeks. The reductions were statistically significantly greater than those receiving placebo at some skin sites.
		30	Glutathione 250 mg capsules		
[113]	A randomized, double-blind, matched-pair, placebo-controlled study	30	A placebo lotion	Subjects applied test lotion to one side of the face and a placebo lotion to the other side twice daily for 10 weeks.	The skin melanin index was significantly lower with GSSG lotion treatment compared with placebo lotion treatment from the first week after the start of the trial through to the end of the study period (10th week).
			Glutathione disulfide 2% lotion		
[114]	A randomized, double-blind, placebo-controlled, parallel, three-arm study	20	Glutathione 250 mg capsule	A capsule was orally taken daily for 12 weeks	Melanin index and UV spots were reduced in the glutathione group and the glutathione disulfide group compared to the placebo group.
		18	Glutathione disulfide 250 mg		
		19	Placebo capsule		
[115]	A placebo-controlled study	16	Placebo	Patients were given 2 intravenous injections (glutathione 1200 mg, ascorbic acid, hydrolyzed collagen, NaCl, and aqua) per week for 6 weeks	After 12 injections of glutathione, 6 of 16 (37.5%) subjects showed significant improvement in skin tone, whereas 3 (18.7%) subjects improved with placebo. After stopping the treatment, this improvement was gradually lost in most patients in 6 months.
		16	Glutathione 1200 mg		
[116]	An open-label, single-arm clinical trial	30	Glutathione 500 mg lozenges	Subjects put one lozenge in the mouth against the inner cheek (buccal mucosa) until completely dissolved every morning for 8 weeks.	There was a significant decrease in melanin indices from baseline to end that became evident as early as 2 weeks. The skin lightening effect was observed both in sun-exposed and sun-protected skin.
[117]	A randomized, double-blind, parallel-group, benchmark- and placebo-controlled trial	32	Placebo	Subjects ingested tablets or capsules daily or twice daily for 12 weeks in a blinded format.	Oral supplementation of cystine plus glutathione induced a significant skin lightening after 12 weeks. This combination also induced a significant reduction in the size of facial dark spots after 6 and 12 weeks. The changes were significantly bigger than those obtained not only with placebo but also with cystine alone or glutathione alone.
		31	Glutathione 250 mg		
		30	Cystine 500 mg		
		31	Cystine 500 mg plus glutathione 250 mg		

**Table 2:** Clinical trials on the skin lightening efficacy of cysteamine.

Literature	Study Format	No. of Subjects	Tested Materials	Treatment	Key Findings
[123]	A randomized, double-blind placebo-controlled study	25	A placebo cream	Cysteamine cream or placebo was applied on the lesions once a day at bedtime over 4 months.	The mean differences between pigmented and normal skin (calculated by Mexameter) were reduced after 2 and 4 months of application of cysteamine cream. At the end of the treatment, the melasma area severity index (MASI) scores were significantly lower in the cysteamine group vs. placebo.
		25	Cysteamine 5% cream		
[124]	A double-blind placebo-controlled study	20	A placebo cream	Cysteamine cream or placebo was applied on the lesions once a day at bedtime throughout the 4-month study period.	Cysteamine cream significantly reduced the degree of pigmentation measured by Mexameter and Dermacatch after 2 weeks compared to the pre-use and placebo-using groups. After 4 weeks, MASI and IGA scores were significantly lower in the cysteamine group compared to the placebo group.
		20	Cysteamine 5% cream		
[125]	A randomized, double-blind clinical study	25	Modified Kligman’s formula (MKF, 4% hydroquinone, 0.05% retinoic acid, and 0.1% betamethasone)	Cysteamine cream was applied once (15 min) daily, and MKF was applied once (whole night) daily over 4 months.	The cysteamine treatment decreased the modified MASI score to a greater degree (32.3%, 51.3%) compared to MKF (23.7%, 42.3%) at 2 and 4 months, respectively, and these differences were strongly statistically significant. The differences between the MKF and cysteamine groups were not statistically significant in IGA assessments at 2 and 4 months.
		25	Cysteamine 5% cream		
[126]	A randomized, double-blind trial	5	Cysteamine 5% cream	Cysteamine cream was applied once (15 min exposure) daily, and hydroquinone cream was applied once daily over 16 weeks.	There was a 19.7% and 39.1% reduction in modified MASI for the cysteamine group and a 39.2% and 33% reduction in the hydroquinone group at 8 and 16 weeks, respectively. The difference between groups was not statistically significant. Hydroquinone cream was better tolerated than cysteamine cream.
		9	Hydroquinone 4% cream		
[127]	A single-blind randomized clinical study	27	Cysteamine 5% cream	Cysteamine 5% cream was applied on the melasma lesions 30 min before bed for 4 months. Tranexamic acid mesotherapy (0.05 mL) was performed every 4 weeks until 8 weeks.	The MASI scores and Dermacatch values were significantly decreased in both cysteamine (2, 4 months) and tranexamic acid mesotherapy groups (4, 8 weeks) compared with baseline. The improvement rates between the two groups were similar (cysteamine for 2 months vs. tranexamic acid mesotherapy for 4 weeks; cysteamine for 4 months vs. tranexamic acid mesotherapy for 8 weeks).
		27	Tranexamic acid (4 mg mL^−1^) mesotherapy		

## References

[1] Slominski A., Kim T.K., Brozyna A.A., Janjetovic Z., Brooks D.L., Schwab L.P., Skobowiat C., Jozwicki W., Seagroves T.N. (2014). The role of melanogenesis in regulation of melanoma behavior: Melanogenesis leads to stimulation of HIF-1alpha expression and HIF-dependent attendant pathways. Arch. Biochem. Biophys..

[2] Slominski R.M., Zmijewski M.A., Slominski A.T. (2015). The role of melanin pigment in melanoma. Exp. Dermatol..

[3] Costin G.E., Hearing V.J. (2007). Human skin pigmentation: Melanocytes modulate skin color in response to stress. FASEB J..

[4] Schiaffino M.V. (2010). Signaling pathways in melanosome biogenesis and pathology. Int. J. Biochem. Cell Biol..

[5] Yamaguchi Y., Beer J.Z., Hearing V.J. (2008). Melanin mediated apoptosis of epidermal cells damaged by ultraviolet radiation: Factors influencing the incidence of skin cancer. Arch. Dermatol. Res..

[6] Boo Y.C. (2020). Emerging Strategies to Protect the Skin from Ultraviolet Rays Using Plant-Derived Materials. Antioxidants.

[7] Rose P.T. (2009). Pigmentary disorders. Med. Clin. N. Am..

[8] Ganju P., Nagpal S., Mohammed M.H., Nishal Kumar P., Pandey R., Natarajan V.T., Mande S.S., Gokhale R.S. (2016). Microbial community profiling shows dysbiosis in the lesional skin of Vitiligo subjects. Sci. Rep..

[9] Spritz R.A., Andersen G.H. (2017). Genetics of Vitiligo. Dermatol. Clin..

[10] Ganceviciene R., Liakou A.I., Theodoridis A., Makrantonaki E., Zouboulis C.C. (2012). Skin anti-aging strategies. Dermatoendocrinology.

[11] Ramos-e-Silva M., Celem L.R., Ramos-e-Silva S., Fucci-da-Costa A.P. (2013). Anti-aging cosmetics: Facts and controversies. Clin. Dermatol..

[12] Zhu W., Gao J. (2008). The use of botanical extracts as topical skin-lightening agents for the improvement of skin pigmentation disorders. J. Investig. Dermatol. Symp. Proc..

[13] Niu C., Aisa H.A. (2017). Upregulation of Melanogenesis and Tyrosinase Activity: Potential Agents for Vitiligo. Molecules.

[14] Pillaiyar T., Namasivayam V., Manickam M., Jung S.H. (2018). Inhibitors of Melanogenesis: An Updated Review. J. Med. Chem..

[15] Maymone M.B.C., Neamah H.H., Secemsky E.A., Vashi N.A. (2018). Correlating the Dermatology Life Quality Index and Skin Discoloration Impact Evaluation Questionnaire tools in disorders of hyperpigmentation. J. Dermatol..

[16] Nautiyal A., Wairkar S. (2021). Management of hyperpigmentation: Current treatments and emerging therapies. Pigment Cell Melanom. Res..

[17] Perez-Bernal A., Munoz-Perez M.A., Camacho F. (2000). Management of facial hyperpigmentation. Am. J. Clin. Dermatol..

[18] Draelos Z.D. (2007). Skin lightening preparations and the hydroquinone controversy. Dermatol. Ther..

[19] Tse T.W. (2010). Hydroquinone for skin lightening: Safety profile, duration of use and when should we stop?. J. Dermatol. Treat..

[20] Boo Y.C. (2021). Mechanistic Basis and Clinical Evidence for the Applications of Nicotinamide (Niacinamide) to Control Skin Aging and Pigmentation. Antioxidants.

[21] Boo Y.C. (2021). Arbutin as a Skin Depigmenting Agent with Antimelanogenic and Antioxidant Properties. Antioxidants.

[22] Kelm R.C., Zahr A.S., Kononov T., Ibrahim O. (2020). Effective lightening of facial melasma during the summer with a dual regimen: A prospective, open-label, evaluator-blinded study. J. Cosmet. Dermatol..

[23] Raposo G., Marks M.S. (2002). The dark side of lysosome-related organelles: Specialization of the endocytic pathway for melanosome biogenesis. Traffic.

[24] Boo Y.C. (2020). Up- or Downregulation of Melanin Synthesis Using Amino Acids, Peptides, and Their Analogs. Biomedicines.

[25] Kim J.H., Seok J.K., Kim Y.M., Boo Y.C. (2019). Identification of small peptides and glycinamide that inhibit melanin synthesis using a positional scanning synthetic peptide combinatorial library. Br. J. Dermatol..

[26] Boo Y.C., Jo D.J., Oh C.M., Lee S.Y., Kim Y.M. (2020). The First Human Clinical Trial on the Skin Depigmentation Efficacy of Glycinamide Hydrochloride. Biomedicines.

[27] Boo Y.C. (2019). Human Skin Lightening Efficacy of Resveratrol and Its Analogs: From in Vitro Studies to Cosmetic Applications. Antioxidants.

[28] Boo Y.C. (2019). p-Coumaric Acid as An Active Ingredient in Cosmetics: A Review Focusing on its Antimelanogenic Effects. Antioxidants.

[29] Cooksey C.J., Garratt P.J., Land E.J., Pavel S., Ramsden C.A., Riley P.A., Smit N.P. (1997). Evidence of the indirect formation of the catecholic intermediate substrate responsible for the autoactivation kinetics of tyrosinase. J. Biol. Chem..

[30] Simon J.D., Peles D., Wakamatsu K., Ito S. (2009). Current challenges in understanding melanogenesis: Bridging chemistry, biological control, morphology, and function. Pigment Cell Melanoma Res..

[31] Olivares C., Solano F. (2009). New insights into the active site structure and catalytic mechanism of tyrosinase and its related proteins. Pigment Cell Melanoma Res..

[32] Thody A.J., Higgins E.M., Wakamatsu K., Ito S., Burchill S.A., Marks J.M. (1991). Pheomelanin as Well as Eumelanin Is Present in Human Epidermis. J. Investig. Dermatol..

[33] Halprin K.M., Ohkawara A. (1966). Glutathione and Human Pigmentation. Arch. Dermatol..

[34] Benedetto J.P., Ortonne J.P., Voulot C., Khatchadourian C., Prota G., Thivolet J. (1981). Role of Thiol Compounds in Mammalian Melanin Pigmentation.1. Reduced and Oxidized Glutathione. J. Investig. Dermatol..

[35] Liu M.J., Prakash C., Nauta A., Siezen R.J., Francke C. (2012). Computational Analysis of Cysteine and Methionine Metabolism and Its Regulation in Dairy Starter and Related Bacteria. J. Bacteriol..

[36] Romero L.C., Aroca M.A., Laureano-Marin A.M., Moreno I., Garcia I., Gotor C. (2014). Cysteine and Cysteine-Related Signaling Pathways in Arabidopsis thaliana. Mol. Plant.

[37] Stipanuk M.H., Dominy J.E., Lee J.I., Coloso R.M. (2006). Mammalian cysteine metabolism: New insights into regulation of cysteine metabolism. J. Nutr..

[38] Mato J.M., Corrales F.J., Lu S.C., Avila M.A. (2002). S-adenosylmethionine: A control switch that regulates liver function. FASEB J..

[39] Conter C., Fruncillo S., Fernandez-Rodriguez C., Martinez-Cruz L.A., Dominici P., Astegno A. (2020). Cystathionine beta-synthase is involved in cysteine biosynthesis and-H2S generation in Toxoplasma gondii. Sci. Rep..

[40] Guggenheim S., Flavin M. (1969). Cystathionine Gamma-Synthase—A Pyridoxal Phosphate Enzyme Catalyzing Rapid Exchanges of Beta and Alpha Hydrogen Atoms in Amino Acids. J. Biol. Chem..

[41] Grillo M.A., Colombatto S. (2008). S-adenosylmethionine and its products. Amino Acids.

[42] Paul B.D., Sbodio J.I., Snyder S.H. (2018). Cysteine Metabolism in Neuronal Redox Homeostasis. Trends Pharmacol. Sci..

[43] Koppula P., Zhuang L., Gan B.Y. (2021). Cystine transporter SLC7A11/xCT in cancer: Ferroptosis, nutrient dependency, and cancer therapy. Protein Cell.

[44] Lo M., Wang Y.Z., Gout P.W. (2008). The x(c)(-) cystine/glutamate antiporter: A potential target for therapy of cancer and other diseases. J. Cell. Physiol..

[45] Mironov A., Seregina T., Shatalin K., Nagornykh M., Shakulov R., Nudler E. (2020). CydDC functions as a cytoplasmic cystine reductase to sensitize Escherichia coli to oxidative stress and aminoglycosides. Proc. Natl. Acad. Sci. USA.

[46] States B., Segal S. (1973). Interrelationship of Glutathione-Cystine Transhydrogenase and Glutathione Reductase in Developing Rat Intestine. Biochem. J..

[47] Bjorn-Yoshimoto W.E., Underhill S.M. (2016). The importance of the excitatory amino acid transporter 3 (EAAT3). Neurochem. Int..

[48] Adelmann C.H., Traunbauer A.K., Chen B., Condon K.J., Chan S.H., Kunchok T., Lewis C.A., Sabatini D.M. (2020). MFSD12 mediates the import of cysteine into melanosomes and lysosomes. Nature.

[49] Poole L.B. (2015). The basics of thiols and cysteines in redox biology and chemistry. Free Radic. Biol. Med..

[50] Bansal A., Simon M.C. (2018). Glutathione metabolism in cancer progression and treatment resistance. J. Cell Biol..

[51] McBean G.J., Aslan M., Griffiths H.R., Torrao R.C. (2015). Thiol redox homeostasis in neurodegenerative disease. Redox Biol..

[52] Lu S.C. (2013). Glutathione synthesis. Biochim. Biophys. Acta-Gen. Subj..

[53] Forman H.J., Zhang H.Q., Rinna A. (2009). Glutathione: Overview of its protective roles, measurement, and biosynthesis. Mol. Asp. Med..

[54] Couto N., Wood J., Barber J. (2016). The role of glutathione reductase and related enzymes on cellular redox homoeostasis network. Free Radic. Biol. Med..

[55] Kennedy L., Sandhu J.K., Harper M.E., Cuperlovic-Culf M. (2020). Role of Glutathione in Cancer: From Mechanisms to Therapies. Biomolecules.

[56] Ballatori N., Krance S.M., Marchan R., Hammond C.L. (2009). Plasma membrane glutathione transporters and their roles in cell physiology and pathophysiology. Mol. Asp. Med..

[57] Zhang H.Q., Forman H.J., Choi J. (2005). gamma-Glutamyl transpeptidase in glutathione biosynthesis. Methods Enzymol..

[58] Gaucher C., Boudier A., Bonetti J., Clarot I., Leroy P., Parent M. (2018). Glutathione: Antioxidant Properties Dedicated to Nanotechnologies. Antioxidants.

[59] Czumaj A., Szrok-Jurga S., Hebanowska A., Turyn J., Swierczynski J., Sledzinski T., Stelmanska E. (2020). The Pathophysiological Role of CoA. Int. J. Mol. Sci..

[60] Genschel U. (2004). Coenzyme A biosynthesis: Reconstruction of the pathway in archaea and an evolutionary scenario based on comparative genomics. Mol. Biol. Evol..

[61] Srinivasan B., Baratashvili M., van der Zwaag M., Kanon B., Colombelli C., Lambrechts R.A., Schaap O., Nollen E.A., Podgorsek A., Kosec G. (2015). Extracellular 4’-phosphopantetheine is a source for intracellular coenzyme A synthesis. Nat. Chem. Biol..

[62] Naquet P., Kerr E.W., Vickers S.D., Leonardi R. (2020). Regulation of coenzyme A levels by degradation: The ‘Ins and Outs’. Prog. Lipid Res..

[63] Pitari G., Malergue F., Martin F., Philippe J.M., Massucci M.T., Chabret C., Maras B., Dupre S., Naquet P., Galland F. (2000). Pantetheinase activity of membrane-bound Vanin-1: Lack of free cysteamine in tissues of Vanin-1 deficient mice. FEBS Lett..

[64] Besouw M., Masereeuw R., van den Heuvel L., Levtchenko E. (2013). Cysteamine: An old drug with new potential. Drug Discov. Today.

[65] Paul B.D., Snyder S.H. (2019). Therapeutic Applications of Cysteamine and Cystamine in Neurodegenerative and Neuropsychiatric Diseases. Front. Neurol..

[66] Qaradakhi T., Gadanec L.K., McSweeney K.R., Abraham J.R., Apostolopoulos V., Zulli A. (2020). The Anti-Inflammatory Effect of Taurine on Cardiovascular Disease. Nutrients.

[67] Wen C.Y., Li F.N., Zhang L.Y., Duan Y.H., Guo Q.P., Wang W.L., He S.P., Li J.Z., Yin Y.L. (2019). Taurine is Involved in Energy Metabolism in Muscles, Adipose Tissue, and the Liver. Mol. Nutr. Food Res..

[68] Miyamoto R., Otsuguro K., Yamaguchi S., Ito S. (2014). Contribution of cysteine aminotransferase and mercaptopyruvate sulfurtransferase to hydrogen sulfide production in peripheral neurons. J. Neurochem..

[69] Cao X., Ding L., Xie Z.Z., Yang Y., Whiteman M., Moore P.K., Bian J.S. (2019). A Review of Hydrogen Sulfide Synthesis, Metabolism, and Measurement: Is Modulation of Hydrogen Sulfide a Novel Therapeutic for Cancer?. Antioxid. Redox Signal..

[70] Stipanuk M.H., Ueki I., Dominy J.E., Simmons C.R., Hirschberger L.L. (2009). Cysteine dioxygenase: A robust system for regulation of cellular cysteine levels. Amino Acids.

[71] Stipanuk M.H. (2020). Metabolism of Sulfur-Containing Amino Acids: How the Body Copes with Excess Methionine, Cysteine, and Sulfide. J. Nutr..

[72] Steinhoff M., Stander S., Seeliger S., Ansel J.C., Schmelz M., Luger T. (2003). Modern aspects of cutaneous neurogenic inflammation. Arch Dermatol..

[73] Slominski A., Tobin D.J., Shibahara S., Wortsman J. (2004). Melanin pigmentation in mammalian skin and its hormonal regulation. Physiol. Rev..

[74] Flaherty K.T., Hodi F.S., Fisher D.E. (2012). From genes to drugs: Targeted strategies for melanoma. Nat. Rev. Cancer.

[75] Serre C., Busuttil V., Botto J.M. (2018). Intrinsic and extrinsic regulation of human skin melanogenesis and pigmentation. Int. J. Cosmet. Sci..

[76] Rzepka Z., Buszman E., Beberok A., Wrzesniok D. (2016). From tyrosine to melanin: Signaling pathways and factors regulating melanogenesis. Postepy Hig. Med. Dosw..

[77] D’Mello S.A., Finlay G.J., Baguley B.C., Askarian-Amiri M.E. (2016). Signaling Pathways in Melanogenesis. Int. J. Mol. Sci..

[78] Yuan X.H., Jin Z.H. (2018). Paracrine regulation of melanogenesis. Br. J. Dermatol..

[79] Ito S., Wakamatsu K. (2008). Chemistry of mixed melanogenesis--pivotal roles of dopaquinone. Photochem. Photobiol..

[80] Kishida R., Saputro A.G., Kasai H. (2015). Mechanism of dopachrome tautomerization into 5,6-dihydroxyindole-2-carboxylic acid catalyzed by Cu(II) based on quantum chemical calculations. Biochim. Biophys. Acta-Gen. Subj..

[81] Sugumaran M. (1991). Molecular Mechanisms for Mammalian Melanogenesis—Comparison with Insect Cuticular Sclerotization. FEBS Lett..

[82] Jara J.R., Aroca P., Solano F., Martinez J.H., Lozano J.A. (1988). The role of sulfhydryl compounds in mammalian melanogenesis: The effect of cysteine and glutathione upon tyrosinase and the intermediates of the pathway. Biochim. Biophys. Acta.

[83] Ito S. (2003). A chemist’s view of melanogenesis. Pigment Cell Res..

[84] Hansson C., Rorsman H., Rosengren E. (1980). 5-S-Cysteinyldopa as a Substrate for Tyrosinase. Acta Dermatol. Venereol..

[85] Wakamatsu K., Ohtara K., Ito S. (2009). Chemical analysis of late stages of pheomelanogenesis: Conversion of dihydrobenzothiazine to a benzothiazole structure. Pigment Cell Melanoma Res..

[86] Ito S., Palumbo A., Prota G. (1985). Tyrosinase-catalyzed conjugation of dopa with glutathione. Experientia.

[87] Carstam R., Hansson C., Lindbladh C., Rorsman H., Rosengren E. (1987). Dopaquinone addition products in cultured human melanoma cells. Acta Dermatol. Venereol..

[88] Benathan M., Virador V., Furumura M., Kobayashi N., Panizzon R.G., Hearing V.J. (1999). Co-regulation of melanin precursors and tyrosinase in human pigment cells: Roles of cysteine and glutathione. Cell. Mol. Biol..

[89] Sanada H., Suzue R., Nakashima Y., Kawada S. (1972). Effect of thiol compounds on melanin formation by tyrosinase. Biochim. Biophys. Acta Gen. Subj..

[90] Kahn V. (1985). Effect of Proteins, Protein Hydrolyzates and Amino Acids on o-Dihydroxyphenolase Activity of Polyphenol Oxidase of Mushroom, Avocado, and Banana. J. Food Sci..

[91] Kermasha S., Goetghebeur M., Monfette A., Metche M., Rovel B. (1993). Inhibitory Effects of Cysteine and Aromatic-Acids on Tyrosinase Activity. Phytochemistry.

[92] Wang S.F., Oh S., Si Y.X., Wang Z.J., Han H.Y., Lee J., Qian G.Y. (2012). Computational prediction of protein-protein interactions of human tyrosinase. Enzym. Res..

[93] Jergil B., Lindbladh C., Rorsman H., Rosengren E. (1984). Inactivation of human tyrosinase by cysteine. Protection by dopa and tyrosine. Acta Dermatol. Venereol..

[94] Tseng T.S., Tsai K.C., Chen W.C., Wang Y.T., Lee Y.C., Lu C.K., Don M.J., Chang C.Y., Lee C.H., Lin H.H. (2015). Discovery of Potent Cysteine-Containing Dipeptide Inhibitors against Tyrosinase: A Comprehensive Investigation of 20 × 20 Dipeptides in Inhibiting Dopachrome Formation. J. Agric. Food Chem..

[95] Hsiao N.W., Tseng T.S., Lee Y.C., Chen W.C., Lin H.H., Chen Y.R., Wang Y.T., Hsu H.J., Tsai K.C. (2014). Serendipitous Discovery of Short Peptides from Natural Products as Tyrosinase Inhibitors. J. Chem. Inf. Model..

[96] Luisi G., Stefanucci A., Zengin G., Dimmito M.P., Mollica A. (2018). Anti-Oxidant and Tyrosinase Inhibitory In Vitro Activity of Amino Acids and Small Peptides: New Hints for the Multifaceted Treatment of Neurologic and Metabolic Disfunctions. Antioxidants.

[97] Lee H.K., Ha J.W., Hwang Y.J., Boo Y.C. (2021). Identification of L-Cysteinamide as a Potent Inhibitor of Tyrosinase-Mediated Dopachrome Formation and Eumelanin Synthesis. Antioxidants.

[98] Kwon B.S., Haq A.K., Pomerantz S.H., Halaban R. (1987). Isolation and sequence of a cDNA clone for human tyrosinase that maps at the mouse c-albino locus. Proc. Natl. Acad. Sci. USA.

[99] Wichers H.J., Recourt K., Hendriks M., Ebbelaar C.E., Biancone G., Hoeberichts F.A., Mooibroek H., Soler-Rivas C. (2003). Cloning, expression and characterisation of two tyrosinase cDNAs from Agaricus bisporus. Appl. Microbiol. Biotechnol..

[100] An S.M., Koh J.S., Boo Y.C. (2010). p-coumaric acid not only inhibits human tyrosinase activity in vitro but also melanogenesis in cells exposed to UVB. Phytother. Res..

[101] Mann T., Gerwat W., Batzer J., Eggers K., Scherner C., Wenck H., Stab F., Hearing V.J., Rohm K.H., Kolbe L. (2018). Inhibition of Human Tyrosinase Requires Molecular Motifs Distinctively Different from Mushroom Tyrosinase. J. Investig. Dermatol..

[102] del Marmol V., Ito S., Bouchard B., Libert A., Wakamatsu K., Ghanem G., Solano F. (1996). Cysteine deprivation promotes eumelanogenesis in human melanoma cells. J. Investig. Dermatol..

[103] Smit N.P.M., VanderMeulen H., Koerten H.K., Kolb R.M., Mommaas A.M., Lentjes E.G.W.M., Pavel S. (1997). Melanogenesis in cultured melanocytes can be substantially influenced by L-tyrosine and L-cysteine. J. Investig. Dermatol..

[104] Qiu L., Zhang M., Sturm R.A., Gardiner B., Tonks I., Kay G., Parsons P.G. (2000). Inhibition of melanin synthesis by cystamine in human melanoma cells. J. Investig. Dermatol..

[105] Chung B.Y., Choi S.R., Moon I.J., Park C.W., Kim Y.H., Chang S.E. (2016). The Glutathione Derivative, GSH Monoethyl Ester, May Effectively Whiten Skin but GSH Does Not. Int. J. Mol. Sci..

[106] Choi H.R., Kang Y.A., Lee H.S., Park K.C. (2015). Disulfanyl peptide decreases melanin synthesis via receptor-mediated ERK activation and the subsequent downregulation of MITF and tyrosinase. Int. J. Cosmet. Sci..

[107] Villarama C.D., Maibach H.I. (2005). Glutathione as a depigmenting agent: An overview. Int. J. Cosmet. Sci..

[108] Dilokthornsakul W., Dhippayom T., Dilokthornsakul P. (2019). The clinical effect of glutathione on skin color and other related skin conditions: A systematic review. J. Cosmet. Dermatol..

[109] Sonthalia S., Jha A.K., Lallas A., Jain G., Jakhar D. (2018). Glutathione for skin lightening: A regnant myth or evidence-based verity?. Dermatol. Pract. Concept..

[110] Yamada M., Ogawa Y., Ikeda T. (1967). Clinical use of synthetic glutathione (Tathione) in various skin diseases. Acta Dermatol.-Kyoto. Engl. Ed..

[111] Han J.Y., Park S.O., Hahm J.H., Kook H.I. (1977). Clinical Effect of Glutathione (Tathione) on Melasma. Korean J. Dermatol..

[112] Arjinpathana N., Asawanonda P. (2012). Glutathione as an oral whitening agent: A randomized, double-blind, placebo-controlled study. J. Dermatol. Treat..

[113] Watanabe F., Hashizume E., Chan G.P., Kamimura A. (2014). Skin-whitening and skin-condition-improving effects of topical oxidized glutathione: A double-blind and placebo-controlled clinical trial in healthy women. Clin. Cosmet. Investig. Dermatol..

[114] Weschawalit S., Thongthip S., Phutrakool P., Asawanonda P. (2017). Glutathione and its antiaging and antimelanogenic effects. Clin. Cosmet. Investig. Dermatol..

[115] Zubair S., Hafeez S., Mujtaba G. (2016). Efficacy of intravenous glutathione vs. placebo for skin tone lightening. J. Pak. Assoc. Dermatol..

[116] Handog E.B., Datuin M.S.L., Singzon I.A. (2016). An open-label, single-arm trial of the safety and efficacy of a novel preparation of glutathione as a skin-lightening agent in Filipino women. Int. J. Dermatol..

[117] Duperray J., Sergheraert R., Chalothorn K., Tachalerdmanee P., Perin F. (2022). The effects of the oral supplementation of L-Cystine associated with reduced L-Glutathione-GSH on human skin pigmentation: A randomized, double-blinded, benchmark- and placebo-controlled clinical trial. J. Cosmet. Dermatol..

[118] Wilkes M., Wright C.Y., du Plessis J.L., Reeder A. (2015). Fitzpatrick Skin Type, Individual Typology Angle, and Melanin Index in an African Population: Steps Toward Universally Applicable Skin Photosensitivity Assessments. JAMA Dermatol..

[119] Adil M., Amin S.S., Mohtashim M. (2018). N-acetylcysteine in dermatology. Indian J. Dermatol. Venereol. Leprol..

[120] Njoo M.D., Menke H.E., Pavel S., Westerhof W. (1997). N-acetylcysteine as a bleaching agent in the treatment of melasma. J. Eur. Acad. Dermatol. Venereol..

[121] Fujiwara Y., Sahashi Y., Aritro M., Hasegawa S., Akimoto K., Ninomiya S., Sakaguchi Y., Seyama Y. (2004). Effect of simultaneous administration of vitamin C, L-cysteine and vitamin E on the melanogenesis. Biofactors.

[122] Ahramiyanpour N., Saki N., Akbari Z., Shamsi-Meymandi S., Amiri R., Heiran A. (2021). Efficacy of topical cysteamine hydrochloride in treating melasma: A systematic review. J. Cosmet. Dermatol..

[123] Mansouri P., Farshi S., Hashemi Z., Kasraee B. (2015). Evaluation of the efficacy of cysteamine 5% cream in the treatment of epidermal melasma: A randomized double-blind placebo-controlled trial. Br. J. Dermatol..

[124] Farshi S., Mansouri P., Kasraee B. (2018). Efficacy of cysteamine cream in the treatment of epidermal melasma, evaluating by Dermacatch as a new measurement method: A randomized double blind placebo controlled study. J. Dermatol. Treat..

[125] Karrabi M., David J., Sahebkar M. (2021). Clinical evaluation of efficacy, safety and tolerability of cysteamine 5% cream in comparison with modified Kligman’s formula in subjects with epidermal melasma: A randomized, double-blind clinical trial study. Ski. Res. Technol..

[126] Nguyen J., Remyn L., Chung I.Y., Honigman A., Wutami I., Mane S., Wong C., Rodrigues M. (2021). Evaluation of the efficacy of cysteamine cream compared to hydroquinone in the treatment of melasma: A randomised, double-blinded, trial. Australas. J. Dermatol..

[127] Karrabi M., Mansournia M.A., Sharestanaki E., Abdollahnejad Y., Sahebkar M. (2021). Clinical evaluation of efficacy and tolerability of cysteamine 5% cream in comparison with tranexamic acid mesotherapy in subjects with melasma: A single-blind, randomized clinical trial study. Arch. Dermatol. Res..

[128] Singh S., Singh U., Pandey S.S. (2011). Increased Level of Serum Homocysteine in Vitiligo. J. Clin. Lab. Anal..

[129] Silverberg J., Silverberg N. (2011). Serum homocysteine is associated with extent of vitiligo vulgaris. J. Am. Acad. Dermatol..

[130] Tsai T.Y., Kuo C.Y., Huang Y.C. (2019). Serum homocysteine, folate, and vitamin B-12 levels in patients with vitiligo and their potential roles as disease activity biomarkers: A systematic review and meta-analysis. J. Am. Acad. Dermatol..

[131] Chen J., Zhuang T., Chen J., Tian Y., Yi X., Ni Q., Zhang W., Song P., Jian Z., Liu L. (2020). Homocysteine induces melanocytes apoptosis via PERK-eIF2alpha-CHOP pathway in vitiligo. Clin. Sci..

[132] Reish O., Townsend D., Berry S.A., Tsai M.Y., King R.A. (1995). Tyrosinase Inhibition Due to Interaction of Homocyst(E)Ine with Copper—The Mechanism for Reversible Hypopigmentation in Homocystinuria Due to Cystathionine Beta-Synthase Deficiency. Am. J. Hum. Genet..

[133] Dey-Rao R., Sinha A.A. (2016). Interactome analysis of gene expression profile reveals potential novel key transcriptional regulators of skin pathology in vitiligo. Genes Immun..

[134] Hwang Y.J., Boo Y.C. (2021). Melanogenesis Promotion by 3-Deazaneplanocin A, a Specific Inhibitor of S-Adenosylhomocysteine Hydrolase, in B16/F10 Melanoma Cells. J. Soc. Cosmet. Sci. Korea.

[135] Denat L., Kadekaro A.L., Marrot L., Leachman S.A., Abdel-Malek Z.A. (2014). Melanocytes as instigators and victims of oxidative stress. J. Investig. Dermatol..

[136] Abbas K., Qadir M.I., Anwar S. (2019). The Role of Melanin in Skin Cancer. Crit. Rev. Eukaryot. Gene Expr..

[137] Smit N.P.M., Nieuwpoort F.A., Marrot L., Out C., Poorthuis B., van Pelt H., Meunier J.R., Pavel S. (2008). Increased melanogenesis is a risk factor for oxidative DNA damage—Study on cultured melanocytes and atypical nevus cells. Photochem. Photobiol..

[138] Lu Y.Y., Tonissen K.F., Di Trapani G. (2021). Modulating skin colour: Role of the thioredoxin and glutathione systems in regulating melanogenesis. Biosci. Rep..

[139] Atallah C., Charcosset C., Greige-Gerges H. (2020). Challenges for cysteamine stabilization, quantification, and biological effects improvement. J. Pharm. Anal..

[140] Khan N.U., Ali A., Khan H., Khan Z.U., Ahmed Z. (2018). Stability Studies and Characterization of Glutathione-Loaded Nanoemulsion. J. Cosmet. Sci..

[141] Demirkol O., Adams C., Ercal N. (2004). Biologically important Thiols in various vegetables and fruits. J. Agric. Food Chem..

[142] Hand C.E., Honek J.F. (2005). Biological chemistry of naturally occurring thiols of microbial and marine origin. J. Nat. Prod..

